# Supplementary material for: Suppressive Effect of Soil Microbiomes Associated with Tropical Fruit Trees on Meloidogyne enterolobii
Source: Microorganisms. 2022 Apr 25;10(5):894. doi: 10.3390/microorganisms10050894 (PMC9144879; doi:10.3390/microorganisms10050894)
Supplement: Supplementary file 1 [file microorganisms-10-00894-s001.zip › Table S2.pdf]

Table S2. Nematode reproduction data for various parameters (per root system) determined for *Meloidogyne enterolobii* population of which eggs and second-stage juveniles (J2) were inoculated on roots of a susceptible tomato 'Moneymaker' and kept in a temperature-controlled glasshouse for 7 weeks.

Treatments	Numbers of eggs and J2/root system		Rf Value[1]	
	First Exp.	Repeat Exp.	First Exp.	Repeat Exp.
**SA1**	3.4[2] (2629[3]) abcd	3.7 (5192) ab	1.31 abc	2.59 abcd
**SA2**	3.4 (2703) abcd	3.8 (7454) bc S[4]	1.35 abc	3.72 cd S
**SA3**	3.3 (2862) abc	3.8 (7844) bc S	1.43 abcd	3.92 d S
**SA4**	3.6 (4489) cdef	3.5 (3850) bc	2.24 bcde	1.92 ab
**SA5**	3.7 (5464) def	3.5 (3949) ab	2.73 cdef	1.97 abc
**SA6**	3.3 (2293) abc	3.5 (4387) ab	1.14 ab	2.19 abcd
**SA7**	3.5 (3839) bcde	3.8 (7315) bc	1.91 abcde	3.65 bcd
**SA8**	3.2 (2052) ab	3.6 (4048) ab	1.02 ab	2.02 abc
**SA9**	3.5 (3762) bcde	3.7 (5967) ab	1.88 abcde	2.98 abcd
**SA10**	3.5 (4099) bcdef	3.7 (6776) abc	2.04 abcde	3.38 abcd
**SA11**	3.7 (6483) ef	3.7 (5213) ab	3.24 ef	2.60 abcd
**SA12**	3.09 (1246) a	3.4 (3270) a S	0.62 a	1.63 a
**SA13**	3.7 (5911) ef	3.5 (3678) ab	2.95 def	1.83 ab
**SA14**	3.3 (2644) abc	3.5 (4418) ab	1.32 abc	2.20 abcd
**The control**	3.9 (8382) f	4.08 (12209) d	4.19 f	6.10 e S
***P* value**	0.000	0.00	0.000	0.000
***F* value**	11.15	150.2	9.976	10.23
**Interaction data: Experiments x treatments**				
***P* value**	0.000		0.000	
***F* value**	5.43		5.47	

[1] Rf = final egg and J2 numbers (*P*f)/initial egg and J2 numbers (*P*i) (Windham & Williams, 1987).

[2] Log (x) transformed value.

[3] Real means.

[4] Significant difference between the same treatments of two experiments (Tukey's Test at $P \leq 0.05$); Lower case letters indicate differences in egg and J2 numbers and reproduction parameters among treatments for each individual experiment, with means in each column followed by the same letter not differing significantly at $P \leq 0.05$;
