# Supplementary figures and images for: Suppressive Effect of Soil Microbiomes Associated with Tropical Fruit Trees on Meloidogyne enterolobii
Source: Microorganisms. 2022 Apr 25;10(5):894. doi: 10.3390/microorganisms10050894 (PMC9144879; doi:10.3390/microorganisms10050894)

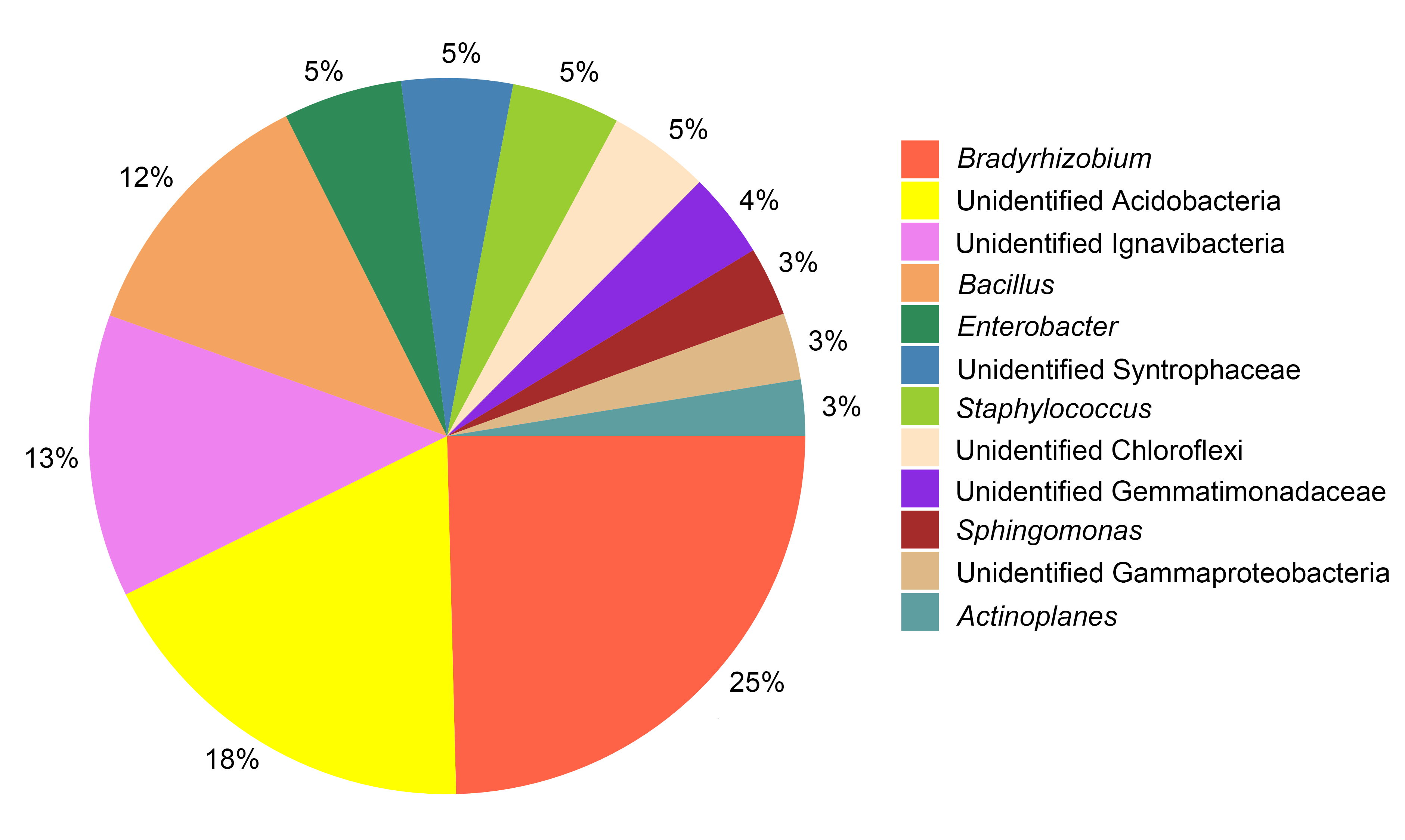

Supplement: Supplementary file 1 [file microorganisms-10-00894-s001.zip › Figure S1.tif]

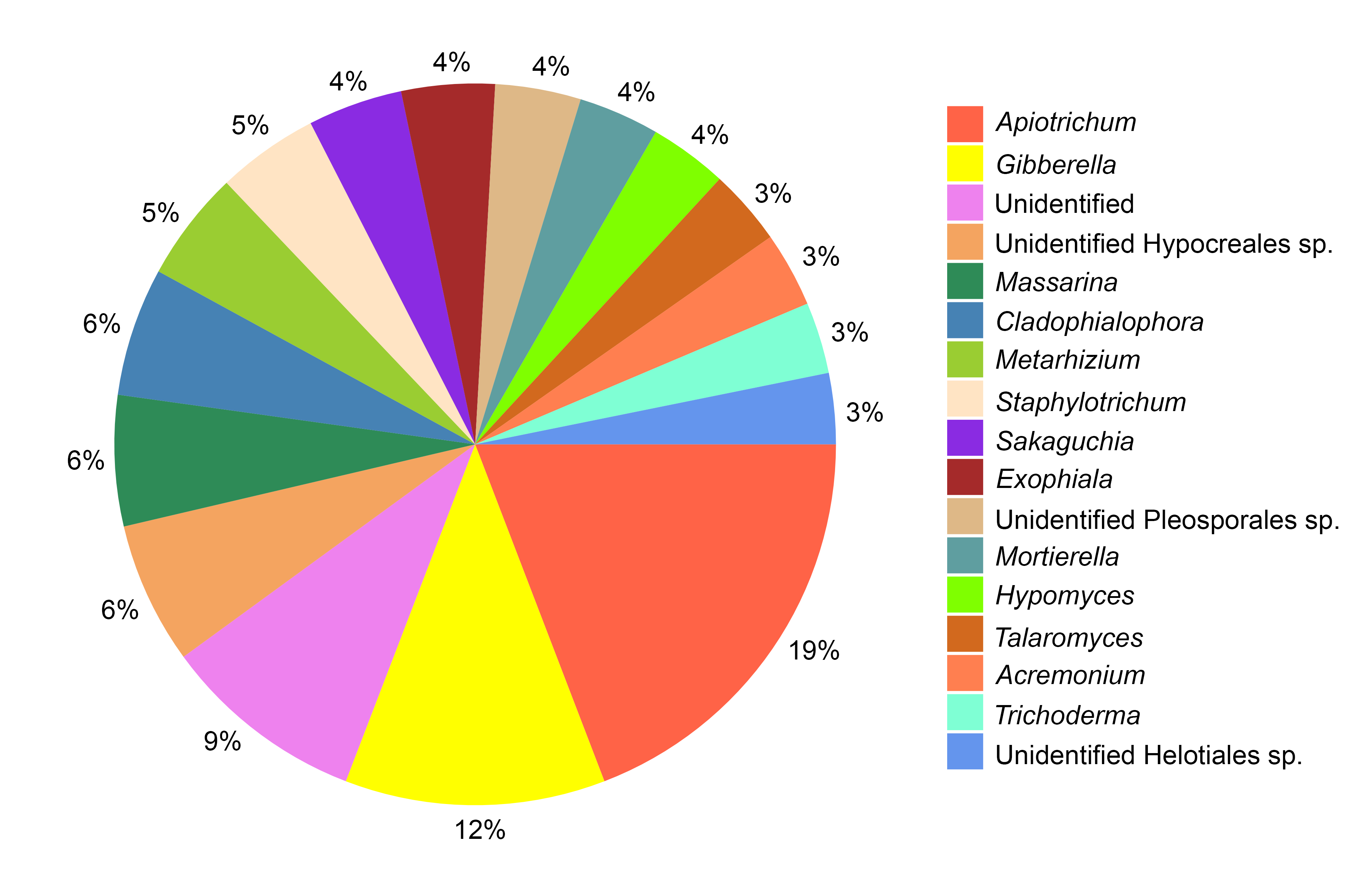

Supplement: Supplementary file 1 [file microorganisms-10-00894-s001.zip › Figure S2.tif]
